# Perinatal risk factors for neonatal asphyxia in Vali-e-Asr hospital, Tehran-Iran

**Published:** 2012-03

**Authors:** Fatemeh Nayeri, Mamak Shariat, Hosein Dalili, Leila Bani Adam, Fatemeh Zareh Mehrjerdi, Afsaneh Shakeri

**Affiliations:** 1 *Maternal, Fetal and Neonatal Research Center, Tehran University of Medical Sciences, Tehran, Iran.*; 2 *Breast Feeding Research Center, Tehran University of Medical Sciences, Tehran, Iran.*

**Keywords:** *Asphyxia*, *Risk Factor*, *Perinatal*

## Abstract

**Background: **Asphyxia is a medical condition in which placental or pulmonary gas exchange is impaired or they cease all together, typically producing a combination of progressive hypoxemia and hypercapnea.

**Objective: **In addition to regional differences in its etiology; it is important to know its risk factors.

**Materials and Methods: **This is a case-control study, all neonates born from May 2002 to September 2005 in Vali-e-Asr Hospital were studied. 9488 newborns were born of which 6091 of the live patients were hospitalized in NICU. 546 newborns were studied as case and control group. 260 neonates (48%) were female and 286 neonates (52%) were male. Among the neonates who were admitted, 182 of them were diagnosed with asphyxia and twice of them (364 newborns) were selected as a control group. The variables consist of; gestational age, type of delivery, birth weight, prenatal care, pregnancy and peripartum complications and neonatal disorders.

**Results: **Our studies showed that 35 (19.2%) patients had mild asphyxia, 107 (58.8%) had moderate asphyxia and 40 (22%) were diagnosed as severe asphyxia. Mean maternal age was 34.23±4.29yr; (range: 23-38 yr); and mean of parity was 2±1.2; (range: 1-8). Risk factors in our study included emergent Caesarian Section, preterm labor (<37w), low birth weight (<2500g), 5 minute Apgar (less than 6), need for resuscitation, nuchal cord, impaired Biophysical Profile, neonatal anemia, and maternal infertility.

**Conclusion:** All risk factors listed above play a role in asphyxia. The majority of these factors are avoidable by means of good perinatal care.

## Introduction

Asphyxia is a medical condition in which placental or pulmonary gas exchange is impaired or they cease altogether, typically producing a combination of progressive hypoxemia and hypercopnea (1). The greatest risk of adverse outcome is seen in newborn infants with fetal acidosis (pH<7), a 5min Apgar score of 0-3, hypoxic-ischemic encephalopathy (altered tone, depressed level of consciousness, seizure) and other multiorgan system malfunctions (2, 3). 

Statistical results show that mortality risk is 15-20% in newborn infants with hypoxic-ischemic encephalopathy (HIE). “It was also shown that 25-30% of survivors were left with permanent neuro developmental abnormalities” (2). The rate of prenatal asphyxia in live births in the western hemisphere is approximately 1-1.5%, this was inversely related to gestational age and birth weight (4). 

The frequency of HIE after birth asphyxia was reported to be 1.4 per 1000 in Iceland (5), 3.8 per 1000 live term births, as of moderate to severe HIE in Australia (6.^7^) and 6.1 of 1000 live term birth in Nepal (8). Maternal illnesses, uteroplacental and fetal factors (1) or fetal and neonatal factors (2, 9) are included as the cause of hypoxic ischemia. In a number of studies the risk factor is categorized into Intrapartum and antepartum (6-8, 10). In recent studies several risk factors were highly associated with HIE. 

These include low birth weight, low Apgar score, low pH and hemoglobin level (5), as well as delivery by unskilled birth attendants, prolonged second stage of labor, birth in nongovernmental hospitals, absence of antenatal care (11), inappropriate antenatal care, post term gestation, vacuum extraction, male sex, and prolapsed (12). 

Asphyxia is a preventable condition and in addition to regional differences in its etiology; it is exceptionally imperative to know its risk factors. For this reason, we have studied risk factors of perinatal asphyxia annually at Imam Khomeini (Vali-e-Asr) Hospital in Tehran from May 2002 to September 2005; in live births.

## Materials and methods

This was a case-control study diagnostic criteria of asphyxia (Inclusion criteria) were: umbilical cord pH<7 or 5 min Apgar<6 or 20 minute Apgar score less than 7, multi organs failure in the first 72 hours or convulsion in the first 24 hours of life. Exclusion criteria were neonates suffering from major congenital anomalies or syndromes. 

Our control group consisted of two live borne; who were born following asphyxiated neonates with no sign of asphyxia or congenital anomalies. They matched with case group for underlying conditions like: maternal age, gravidity and demographic factors. Hypoxic ischemic encephalopathy is an important criterion of asphyxia. The clinical spectrum of HIE (Hypoxic Ischemic Encephalopathy) is described as mild, moderate and severe; based on Sarnat and Sarnet stages of HIE (13). 

The variables consist of gestational age, type of delivery, birth weight, prenatal care, pregnancy complication such as preeclampcia, eclampcia, oligo hydramious, poly hydramnious, gestational diabetes mellitus, trauma, major surgery, infertility and impaired biophysical profile; maternal systemic diseases (like chronic renal failure, glumerulonephritis, renal transplantation, coronary artery diseases, cardiomyopathy and congestive heart failure); perinatal complications like premature rupture of membrane, placenta previa, placental abruption and umbilical cord prolapse, nuchal cord and IUGR (-2SD of growth chart adjusted for gestational age); Peripartum complications included prolonged second stage of labor, dystocia, malpresentation of fetus and need for resuscitation and neonatal disorders such as pneumonia, pneumothorax, congestive heart failure, and severe anemia. 

Pregnancy complication, maternal systemic diseases, perinatal and peripartum complications were approved by perinatologists. Neonatal disorders were approved by neonatologists. In this study preterm delivery was defined as gestational age less than 37 weeks. Likewise birth weights were divided to low birth weight (LBW) (BW<2500g), normal birth weight (2500g ≤ BW < 4000g) and macrosomia (BW>4000 g).Complete prenatal care is defined as greater than 6 prenatal visits during pregnancy. Umbilical cord arterial blood gases were done in all of our suspicious neonates for confirmation of diagnosis. 

Data was collected retrospectively based on prepared questionnaires. The questionnaire is based on the variables considered in previous studies. In this research period 9488 newborns were born, of which 6091 of the patients were hospitalized in NICU. Among the neonates who were admitted into the hospital, 182 of them were diagnosed with asphyxia and 364 newborns were selected as control group. Throughout delivery the fetus was monitored every 15 minutes by a specialist. 

Oxytocin infusion and amniotomy were used for some delivery induction. Neonatal resuscitation was done by an expert team with comprehensive and complete equipments. It should be mentioned that this research was under authorization of Tehran University of Medical Sciences Ethic Committee. 

**Statistical analysis**

The recorded data was based on SPSS version 15; furthermore descriptive statistics were extracted with absolute or partial frequency, mean and standard deviation. Analytic statistics were extracted with X^2^, Anova test, logistic regression and Odds ratio.

## Results

In this research the files of 546 neonates were studied in two groups, 182 neonates had asphyxia (case group) and 364 neonates were selected as the control group. In case group, 35 (19.2%) had mild, 107 (58.8%) moderate and 40 (22%) were diagnosed as severe asphyxia. Mean maternal age was 34.23±4.29 yr; (range: 23-38 yr); and mean of parity was 2±1.2; (range: 1-8). 260 neonates (48%) were female and 286 neonates (52%) were male. Assessments of demographic and underlying features between two groups are shown in table I. There was no significant difference between two groups. 

Thus two groups were equally matched. In table II probable risk factors between two groups were compared based on logistic regression analysis. Risk factors of our study were the following: emergent Caesarian Section, preterm labor (<37w), birth weight< 2500g, 5 minute Apgar less than 6 , need for resuscitation, nuchal cord, impaired biophysical profile, neonatal anemia, and infertility and its treatment. 

**Table I:** Comparison of demographic features and maternal underlying diseases between case and control groups

	**Case**	**Control**	**p-value**
Female n (%)	81 (44.5)	179 (49)	0.30
Male n (%)	101 (55.5)	185 (51)	
Parity{mean ±SD}	1.83±1.29	2.08±1.25	0.029
Maternal. Age (mean± SD)	32.99±4.43	35.85±3.16	0.35
Maternal hypotension n (%)	4 (2)	4 (1)	0.31
Maternal autoimmune disease n (%)	7 (4)	10 (3)	0.48
Maternal infectious disease n (%)	10 (5.5)	13 (3.5)	0.29
Maternal cardio vascular disease n (%)	7 (4)	16 (4)	0.76
Maternal renal disease n (%)	7 (4)	1 (0.3)	0.47
Maternal endocrine disease n (%)	21 (10)	44 (12)	0.55
Maternal malignancies n (%)	1 (0.7)	0	0.15
Maternal hematologic disease n (%)	11 (6)	10 (3)	0.06

**Table II:** Comparison of probable risk factors between case and control groups

**Factor**	**Case**	**‍** **Control**	**p-value**	**OR**	**CI/ 95%**
BWT<2500 gr	148 (81)	169 (46)	0.017	3.13	1.23-7.99
Emergent C/S	37 (20)	11 (3)	0.0001	28.50	6.16-131.77
GA<37 W	156 (86)	188 (52)	0.046	2.57	1.01-6.53
GA<35 W	133 (73.1%)	72 (19.8%)	0.000	11.0	7.2-16.7
Incomplete perinatal care	6 (3)	2 (0.5)	0.99	2.87	0.1-8.4
Contraindicated drug usage	30 (16)	74 (20)	0.20	0.42	0.11-1.59
IUGR	32 (18)	78 (21)	0.28	0.56	0.19-1.63
Preeclampsia / Eclampsia	39 (21)	49 (13)	0.95	0.96	0.29-3.12
GDM ^*^	17 (5)	6 (3)	0.99	1.68	54
Need for resuscitation	175 (96)	57 (16)	0.0001	108.40	29.74-395.07
Impaired BPP	6 (3)	2 (0.5)	0.027	197.17	1.80-215.14
Nuchal cord	8 (4.5)	4 (1)	0.007	40.95	2.78-60.31
Dystocia	65 (36)	76 (21)	0.047	19.87	1.06-37.21
PROM	67 (33)	123 (34)	0.61	4.55	0.01-1.67
Fatal hydropse	2 (1)	0	0.31	3.08	0.34-27.63
Infertility and it's treatment	49 (27)	29 (8)	0.003	5.79	1.85-18.13
Placental disorders	33 (18)	37 (10)	0.70	0.31	0.01-135.5
Neonatal anemia	68 (37)	55 (15)	0.01	2.98	1.2-7.40
Maternal thyroid disorder	12 (7)	7 (2)	0.60	8.25	0.92-73.35

## Discussion

Since diagnosis of mild asphyxia is extremely difficult, the largest part of our study was based on moderate to severe cases. By studying all asphyxiated newborns we surprisingly found that 80% of our cases had moderate to severe hypoxic ischemic encephalopathy. In view of the fact that clinical manifestations of mild asphyxia are quite similar to prematurity, most of the studies were made on term newborns (6-8). 

However Vali-e-Asr Hospital is a referral hospital, Almost 50% of neonates are premature or low birth weight. In consequence, our study was done on all neonates (term and preterm). We showed that in case group the rate of prematurity was significantly higher than the control group, even though Butt *et al* reported no co-relation (11). Although in numerous studies, asphyxia was more prevalent in male than female (6, 7, 12), no other significant differences was noticed between male and female in our study. In case group, emergent Caesarian section was significantly more than control group similar to Ellis *et al* study (8) but Badavi *et al* reported that Caesarian section is insignificantly more in case group (6, 7). In our study, 80% of mothers were in the appropriate age category for pregnancy (20-35 yr), while 10% were over 35 years and 10% were younger than 20 years.

Even though some studies indicate that risk of asphyxia is significantly higher in maternal age less than 20 years or more than 35 years (10), no significant distinction was observed in these particular age groups in our study. Our findings confirmed that thyroid disorders were not significantly affective in the case group although Badvai *et al* reported maternal thyroid disorders increase the risk of asphyxia (6, 7) and Ellis *et al* studies showed that only maternal hypothyroidism is associated with neonatal HIE (8). 

In this study, 5 min Apgar< 6, impaired BPP, maternal infertility and its treatment, nuchal cord, shoulder dystocia, and neonatal anemia were considerably more noticeable in case group. Our study showed that 5 minute Apgar less than 6,low birth weight and neonatal anemia are significantly more in case group similar to Palsdottir *et al* study (5). In our case group infertility and its treatment and nuchal cord are significantly more than control group, similar to Badavi *et al* study (6, 7). 

Butt *et al* study showed that delivery by unskilled birth attendant, birth in nongovernmental hospital as well as absence of antenatal care were risk factors (11), while our study was done in one governmental, educational center. Futrakul *et al* described risk factors of asphyxia as 5 min Apgar less than 6 and prolapsed cord similar to our study (12). 

## Conclusion

Our study showed that, emergent Cesarean section, preterm labor (<37 w), birth weight lower than 2500g, need for resuscitation, nuchal cord, impaired biophysical profile, neonatal anemia and maternal infertility and its treatment are risk factors of asphyxia. The majority of these factors may be manageable by means of good prenatal care.
